# Sulphate of Quinine in Congestive Fever

**Published:** 1841-11

**Authors:** 


### SULPHATE OF QUININE IN CONGESTIVE FEVER.

Dr. Preston Capshaw, of Madison county, Ala., in a letter of the 30th of September, writes as follows: "I am now living in one of the sickliest spots in North Alabama, where I have learned much on the treatment of fever. We have all grades, from the mildest to the most malignant. The routine practice of many of our physicians, consists in giving large doses of calomel or —— pills, so as freely to evacuate the bowels, to be followed in the first intermission or remission, by liberal doses of quinine. Venesection and the cold dash are seldom resorted to, and when the latter is applied, it is too often without discretion, whereby mischief results to the patient. Emetics, sinapisms and blisters are, also, occasionally used, but the *sine qua non* with the gentleman of whose practice I am speaking, is to disgorge the liver by means of active mercurial cathartics. This method succeeds very well in ordinary cases; but where much congestion is present, or there is a great tendency to it, I think I have seen manifest injury and even death, follow as its consequence. Under the influence of this conviction I was led to pursue a different practice, and the results have been such as to assure me, that in the congestive, if not in other forms of autumnal fever, cathartics are not only unnecessary, but contra-indicated and injurious.

In congestive fever, as you know, there is a great influx of blood upon the viscera, with cold extremitias, prostration of strength, &c., indicating the patient to be in a chill. This state is followed, sooner or later, for the few first paroxysms, by a partial reaction. My plan in the treatment of such cases, is simply this: when the patient is cold, I direct all my efforts to getting him warm. My means are the cold dash and warm bath alternately, and frictions with mustard, ammonia, &c. When I get them warm, or there is even partial evidence of reaction, I give twenty grains of sulphate of quinine, and repeat it every hour, till four or five doses have been administered. The medicine sometimes produces disagreeable symptoms, such as deafness, blindness, &c.; but I have invariably found my patient better of his fever the next day, after which he continued to mend. When the "fever is broke," if he should be costive, it is well, or, perhaps, in some cases, necessary to administer a laxative, though I find that the great destroyer "black bile" may flow off *spontaneously*, as the patient recovers. I must state, however, that I have as yet pursued this practice in only five decidedly well-marked and violent cases. In two of them I gave no purgative whatever; in two, whose bowels were tardy, I gave castor oil, in one, rhubarb and magnesia. All got well."

